# Gas as medicine: the case for hydrogen gas as a therapeutic agent for critical illness

**DOI:** 10.1186/s40635-025-00798-w

**Published:** 2025-09-12

**Authors:** Lakhmir S. Chawla

**Affiliations:** Department of Medicine, Veterans Affairs Medical Center, 3550 La Jolla Village Drive, San Diego, CA USA

## Abstract

Molecular hydrogen gas (HG), administered through inhalation or as hydrogen-rich fluids (HRF), has demonstrated antioxidant, anti-inflammatory, antiapoptotic, cytoprotective, and beneficial mitochondrial effects in critical illness. Preclinical studies and human clinical studies consistently endorse hydrogen gas as safe, with mechanisms of action linked to vital molecular pathways, such as reductions in both oxidative stress and inflammation with beneficial effects on mitochondria. In preclinical studies, HG has been shown to improve outcomes in conditions such as sepsis, acute lung injury, hepatic injury, pancreatitis, cardiac arrest, traumatic injury, acute kidney injury, and brain injury. HG has been given to human subjects across multiple disease states and has a good safety profile with encouraging clinical effects. Given its accessibility, safety, and low-cost, hydrogen gas therapy should be assessed in adequately powered clinical trials in critical illness.

## Introduction

Hydrogen gas (HG) as a therapy to treat oxidative stress was shown to have potential clinical effects by Ohsawa et al [1]. In this study published in Nature Medicine, Ohsawa and colleagues demonstrated that hydrogen gas is effective at neutralizing hydroxyl radicals and other reactive oxygen species while protecting cells without undermining cell metabolism or signaling [1]. Since low level reactive oxygen species (ROS) signaling is important for normal cellular function, full suppression of ROS may be harmful or otherwise alter normal intracellular signaling [2]. Ohsawa et al. induced acute oxidative stress via three independent methods and found that HG reduced hydroxyl radical but did not react with ROS that possess physiologic roles. Like oxygen, hydrogen gas is small and uncharged, allowing it to cross cell membranes rapidly, thus conferring a large volume of distribution and good end-organ delivery [3]. In addition, HG is shown to pass into the blood brain barrier rapidly and enter cells efficiently allowing the targeting of the mitochondria and other intracellular organelles [3].

Subsequent studies have confirmed that HG can be delivered as hydrogen rich water (HRW) orally, hydrogen rich saline (HRS) as an intravenous infusion, or as inhaled hydrogen gas (iHG) [4]. Across multiple injury models HG has demonstrated antioxidant, antiapoptotic, anti-inflammatory, and cytoprotective capabilities [4]. In preclinical models, HG therapy has been effective in injury models of the brain, heart, lung, liver, kidney, skin, and in vascular disease. Randomized controlled trials of HG have shown promise is Parkinson’s disease, ischemic stroke, cardiac arrest, exercise recovery, and skin inflammation [4].

The human experience with a high percentage of inhaled hydrogen gas for extended periods of time is extensive and is largely derived from the diving literature. At increased atmospheric pressure (e.g. scuba diving), nitrogen can cause narcosis and divers need to utilize gas mixtures wherein nitrogen is replaced with either helium (Heli-Ox) or hydrogen (Hydro-Ox) [5]. As such, multiple divers and marine researchers have had exposure to Hydro-Ox with a hydrogen percentage in excess of 75% for months without any reported adverse events [6].

The simple hypothesis is that HG may effectively and safely mitigate ROS thereby decreasing both tissue injury, oxidative stress, and inflammation in critical illness thereby leading to improved clinical outcomes.

## Mechanism of action

In basic terms, the primary mechanism of action of HG is as a rapid and effective antioxidant, easily diffusing into cells and organelles like mitochondria to help regulate oxidative stress, inflammation, and apoptosis [7–10]. In addition, HG effectively passes through the blood–brain barrier (BBB); thus, conferring potential as an ideal CNS therapeutic [4]. The proposed redox reactions of HG with known reactive oxygen species (ROS) is shown in Table 1 [1, 3, 11]. There are no known enzyme(s) that specifically neutralize hydroxyl radical (OH^−^) since this ROS nonselectively reacts with the nearest nucleophilic molecule that it encounters [3, 4]. However, HG is shown to be a novel reducing agent that can mitigate OH and peroxynitrite (ONOOO^−^) without disrupting normal physiologic function [1, 3, 4, 12]. Interestingly, the biologic and antioxidant effects of HG remain present even after hydrogen has been cleared, which suggests that the observed salutary effects may be due to antioxidant signal modulation and other effects [4, 13–15].

**Table1:**
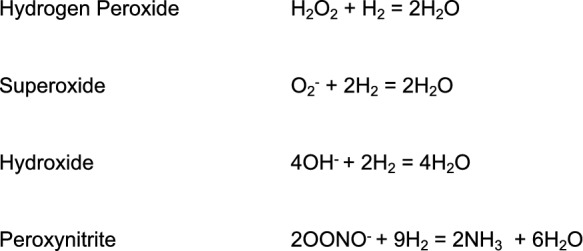
Proposed redox reactions of hydrogen gas with reactive oxygen species

Preclinical studies indeed demonstrate that HG activates the antioxidant response element (ARE) via the nuclear factor erythroid-2 related factor (NRF2) [16–20]. NRF2 is a critical factor in the regulation of oxidative stress. When an organism is challenged with ROS or other pathology, NRF2 is translocated to the nucleus where it binds to the ARE which then activates over 200 genes associated with antioxidant protective response including: glutathione, heme oxygenase-1 (HO-1) and thioredoxin [21, 22].

Beyond the antioxidant response, HG also appears to function as a mitochondrial support molecule, protecting mitochondria from damage, repairing mitochondrial injury, and promoting mitochondrial function [8, 16, 17, 23, 24].

The precise mechanism by which hydrogen gas nourishes and protects mitochondria is not clear. Excessive mitochondrial fission can disrupt energy production and cellular homeostasis [25]. Mitochondrial fission is the process of mitochondrial splitting, but excessive mitochondrial fission can lead to mitochondrial dysfunction, oxidative stress, and fragmentation of the mitochondrial network [26]. Other studies indicate that in sepsis, heightened mitochondrial fission leads to mitochondrial dysfunction, contributing to inflammation and tissue damage, particularly in organs like the lungs. These preclinical studies suggest that HG helps by reducing this excessive fission, thereby protecting mitochondrial function and mitigating sepsis-induced injury [25, 27–29].

Other studies find that HG induces endogenous antioxidant systems [10, 18, 23]. For example, Fan et al. demonstrate that hydrogen-rich saline inhibits lung injury by activating endogenous antioxidant systems through the NF-κB/NLRP3 signaling pathway, reducing oxidative stress and mitigating local Fenton reaction(s) responsible for OH production [10, 30]. In preclinical studies, HG inhalation mitigated oxidative stress and NLRP3 pyroptosis and HG is shown to decrease both the degree of mitochondrial swelling and loss of mitochondrial membrane potential while also preserving mitochondrial cytochrome c content [9, 31]. In aggregate, HG antioxidant effects initiate downstream mitigation of inflammation (e.g. IL-6) and is also associated with initiation of anti-inflammatory pathways (e.g. IL-10) [18, 32].

Another documented mechanism of action of HG is its impact on inflammation. These effects could be due to knock-on impacts of the mitigation of ROS and antioxidant activation or could represent other pathways that are distinctly modulated by HG. Preclinical studies show that HG can reduce the infiltration of leukocytes by downregulating adhesion molecules and chemokines, leading to less inflammation and potentially less tissue damage in many critical conditions [8–10, 20, 32]. Wang and colleagues demonstrate that HG in the form of hydrogen-rich saline inhibits the activation of NF-κB, thereby reducing pro-inflammatory cytokines [33]. This suggests that HG may provide a protective, anti-inflammatory effect by targeting a key regulator of inflammation at the molecular level. Studies of LPS-induced inflammation show that HG dose-dependently attenuates elevated pro-inflammatory cytokines while simultaneously increasing the levels of anti-inflammatory cytokines following LPS stimulation [34, 35]. The aggregate effects of the mechanism of action (MOA) outlined above result in less apoptosis and a decrease of excess autophagy [4].

## Hydrogen gas for neurologic injury

HG is an attractive therapeutic modality for brain injury because of the multimodal MOA and its ability to rapidly pass through the BBB [36]. A multitude of preclinical studies of traumatic brain injury, ischemic stroke, ischemia–reperfusion injury, intracerebral hemorrhage, subarachnoid hemorrhage, and neuro-degenerative disease have shown potential efficacy [36]. Che et al. explored how HG’s ability to act on oxidative stress, inflammation, and apoptosis—all common in TBI, stroke, and neurodegenerative diseases—suggest therapeutic potential in mitigating the damage caused by brain injuries, improving recovery, and reducing the long-term effects of these conditions [36].

**Fig.1:**
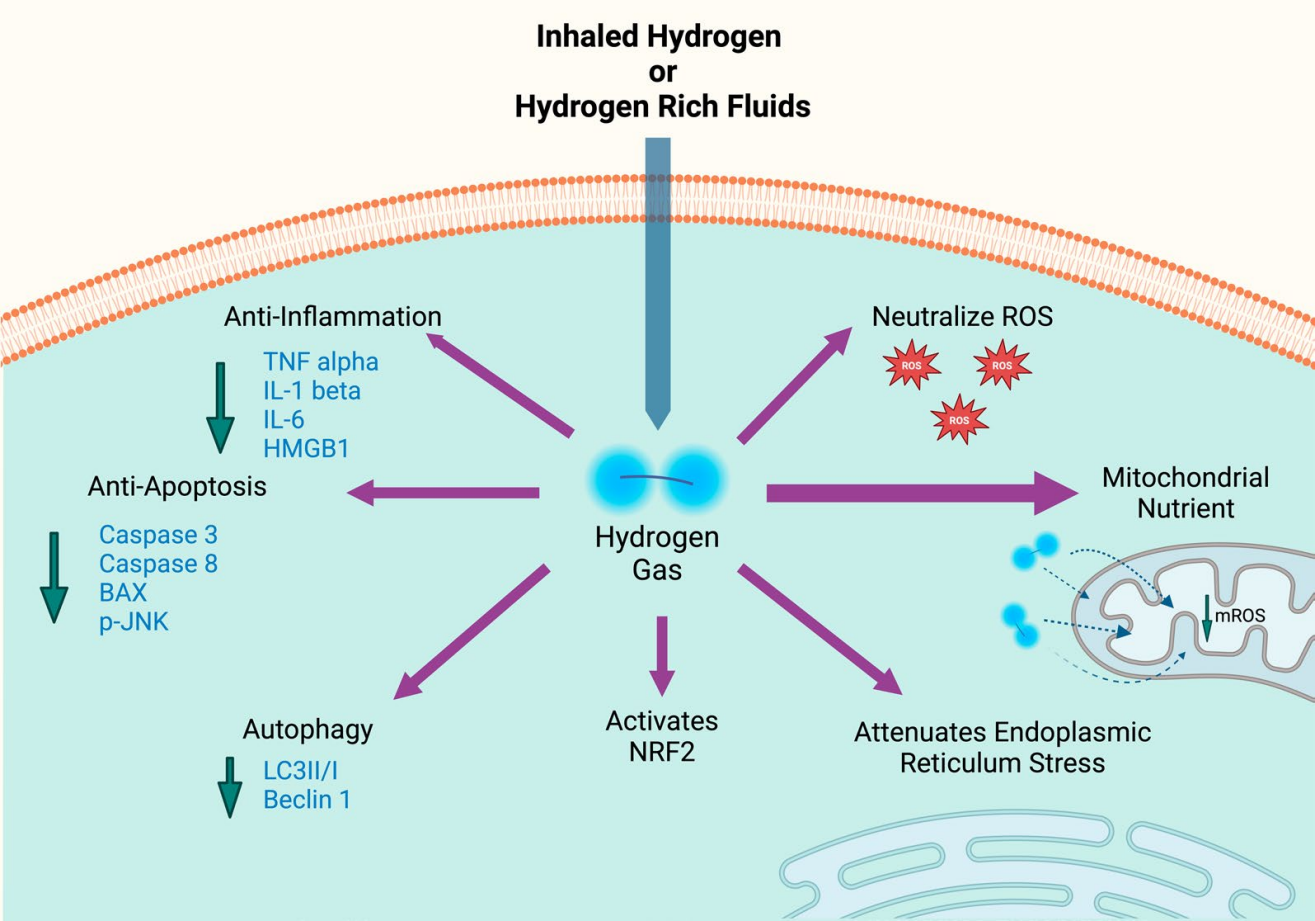
Multimodal effects of hydrogen gas. Hydrogen gas (HG) has multiple potential impacts at the cellular level. HG neutralizes ROS, activates NRF2, and attenuates ER stress. In addition, HG is a mitochondrial nutrient and anti-apoptosis and anti-inflammatory effects

These studies reviewed by Che et al. confirm the protective effects of HG via the mechanisms shown in Fig. 1. Notably, Eckermann and colleagues showed that HG attenuated cerebral edema after surgically induced brain injury [37]. Based on these findings and others, multiple clinical RCTs have been initiated, including trials for neuroprotection after cardiac arrest, ischemic stroke, and Parkinson’s disease [36]. A small Phase 2 study found HG therapy improved scores on the movement disorder scale, specifically the Unified Parkinson’s Disease Rating Scale (UPDRS), suggesting a positive impact on motor function. A larger follow-up study confirmed the safety of HG therapy, but failed to confirm the benefit [38]. For cardiac arrest, a large Phase 2 study of immediate HG after cardiac arrest was stopped early due to the COVID-19 pandemic. However, the results of abbreviated trial showed a tend toward improved survival and neurologic outcome [39].

One pilot study evaluated the safety and neuroprotective effects of HG inhalation for ischemic stroke, finding that treatment was not only safe, but that patients who received the therapy showed a reduction in infarction size and improved clinical outcomes compared to the placebo group [40].

Thus, in various clinical studies of neurologic disease, HG therapy is shown to maintain a strong safety profile with the potential to significantly improve clinical outcomes.

## Hydrogen gas for ARDS/lung injury

Inhaled HG is an ideal therapeutic candidate drug for lung injury as the hydrogen molecules can easily diffuse into the alveoli, endothelial cells, and then the bloodstream. Multiple preclinical studies in acute lung injury (ALI), COVID-19, pneumonia, asthma, chronic obstructive pulmonary disease (COPD), pulmonary arterial hypertension (PAH), and pulmonary fibrosis have demonstrated the salutary effects of inhaled HG [41]. For COVID-19, Guan and colleagues performed an open-label nonrandomized trial of 90 patients with hospital admitted COVID-19 [42]. The intervention was H_2_–O_2_ admixture (66.6% hydrogen–33.3% oxygen) at 6 L/min via nasal cannula. This study showed improved disease severity at Day 2 and Day 3, contributing to the Chinese CDC’s recommendation of H_2_–O_2_ therapy for the treatment of COVID-19 [43].

Multiple trials have been initiated, including a small, randomized study of 50 patients post-acute COVID-19, which showed that after a 14 day intervention with inhaled HG, participants showed improved 6 min walk tests, FVC, and FEV [44]. Clinical studies have shown that inhaled HG is a safe and promising treatment option for both COPD and interstitial lung disease (ILD), improving lung function and outcomes, particularly in early-stage patients [45, 46]. Because inhaled HG can be blended into standard ventilation devices, patients with pulmonary disease, particularly those with ALI, represent good candidate cohorts for HG therapy.

## HG for cardiac injury

Preclinical studies demonstrate the utility of HG therapy in various models of cardiac injury, including radiation-induced cardiac injury, chronic cardiac hypoxia models, ischemia reperfusion injury (IRI), and atherosclerosis [47]. The IRI studies are notable, as therapies to reduce infarct size in patients with acute myocardial infarction are associated with improved outcomes [48].

Aside from rapid and effective reperfusion, therapeutic agents to minimize infarct size and/or subsequent left ventricle (LV) remodeling are lacking. Preclinical studies of cardiac IRI, specifically myocardial infarction (MI) models, demonstrate that inhaled HG indeed reduces infarct size [47]. Clinical studies have also shown promise for HG therapy. An open-label RCT in patients with STEMI after percutaneous coronary intervention (PCI) demonstrated that HG therapy was feasible, safe, and led to improved LV remodeling and function [49].

## HG for liver injury and pancreatitis

Liver injury can occur via multiple pathways and preclinical studies show that HG holds therapeutic potential across a myriad of liver injury models [50]. Animal studies show that HG can mitigate acetaminophen-induced liver injury, obstructive jaundice, hepatic IRI, carbon tetrachloride liver injury and steatohepatitis [50]. Clinical studies of fatty liver disease have shown that HG therapy is safe and associated with improved liver function tests [51].

Acute pancreatitis, an inflammatory disease mediated by damage to acinar cells, triggers systemic inflammation that often affects multiple organs [35]. Interactions between cytokines and oxidative stress significantly contribute to the amplification of uncontrolled inflammatory response [35]. Preclinical studies of inhaled HG document its capacity to ameliorate the damaging effects of inflammation and oxidative stress in acute pancreatitis [35, 52]. Clinical studies of HG for the treatment of pancreatitis have not yet been conducted.

## Hydrogen gas for AKI

The use of HG for both acute and chronic kidney disease has been assessed in multiple preclinical studies with results that show salutary effects consistent with the mechanism of action outlined in Fig. 1 [53]. Multiple preclinical studies demonstrate the potential for HG as a safe and effective treatment of AKI induced by IRI, rhabdomyolysis, nephrotoxin, and ureteral obstruction [53]. A study by Cheng et al. highlights HG’s ability to reduce oxidative stress, inflammation, and apoptosis, which are all critical factors in kidney injury. In addition, they show how HG therapy leads to improved renal function and protection of kidney tissues through its antioxidant properties [53].

In addition to AKI and CKD, HG has been delivered via both peritoneal and dialysate fluids, representing a novel form of HRF delivery. Clinical studies of HG-enriched dialysate in patients receiving chronic hemodialysis has shown to be safe with promising clinical results [54]. In a prospective observational study by Nakayama et al., HG-treated patients showed improvements in their prognosis, including better survival rates and reduced inflammation markers [54]. With no adverse effects reported, this study indicates HG-enriched dialysate could be a beneficial adjunctive therapy for chronic dialysis patients [54].

## Hydrogen gas for sepsis

Sepsis remains a vexing syndrome with inadequate therapeutic options. There have been dozens of preclinical studies that demonstrate potential efficacy of HG therapy via inhalation and HRFs in the treatment of sepsis [55]. In these various investigations, HG influences several key pathways, including anti-inflammatory, anti-oxidant, anti-apoptosis, and anti-shock mechanisms, as illustrated in Fig. 1 [55]. In addition, HG appears to increase autophagy and thus reduces NLRP3 inflammasome activation, which has shown to decrease inflammation, improve mitochondrial function, and protect against organ damage during sepsis [23]. A study conducted by Yu et al. demonstrates how HG helps alleviate physiologic barrier dysfunction and capillary leak by reducing endothelial cell injury and decreasing vascular permeability [56]. Through its antioxidant and anti-inflammatory properties, HG stabilizes the endothelial barrier, thus preventing excessive fluid leakage from capillaries and mitigating tissue edema [55]. Importantly, these therapeutic effects result in better maintenance of vascular integrity and improved outcomes in sepsis [55].

## Potential use of hydrogen in extracorporeal therapies

Another opportunity for HG is the use of extracorporeal systems that incorporate an oxygenator in the blood circuit, such as extracorporeal oxygenation (ECMO) and bypass surgery. Preclinical studies in rat models show that HG can cross the oxygenator membrane, allowing it to reach the bloodstream and exert therapeutic effects [57, 58]. ECMO, commonly used in critically ill patients, induces significant oxidative stress and systemic inflammation due to the interaction of blood with artificial oxygenation membranes and blood pumps [57, 58]. By incorporating HG into ECMO, researchers have observed promising reductions in oxidative stress and inflammation, thereby supporting its potential use as an adjunct therapy in patients requiring an EC circuit [57, 58].

In the above-mentioned preclinical study by Yin et al., HG administration during prolonged cardiac arrest using ECMO led to improved survival rates and reduced cardiac damage, highlighting its potential for enhanced postprocedure outcomes in cardiac bypass surgery [57]. Given the preclinical effects of HG, the addition of HG to cardiac bypass surgery would likely result in better post-procedure cardiac function and recovery, improved pulmonary function, and less AKI. These findings emphasize the therapeutic potential of HG in minimizing complications associated with extracorporeal systems [57, 58].

## Conclusions

HG treatment delivered by inhalation and/or HRF have been shown promise across a wide array of critical conditions, such as organ injury, sepsis, ARDS, and AKI. Given the expansive safety experience with inhaled hydrogen in the marine diving literature and the pleiotropic effects of inhaled hydrogen it is logical that the initial focus in critical care should ALI. The primary reason is that inhaled hydrogen would directly impact the alveoli, Type I and Type II alveolar cells, and would rapidly diffuse into the pulmonary capillaries. For other uses of HG, delivery of HG requires delivery into the bloodstream with delivery to distant tissues via capillaries.

HG therapy is available via electrolysis of water, it is widely available, inexpensive, easily administered, and does not appear to have any adverse events. Given the lack of therapeutic options for many patients with severe critical illness, clinical trials should be initiated to fully explore the therapeutic benefits of hydrogen gas in these life-threatening conditions.

## Data Availability

Author has access to all data for this manuscript. There are no original data presented—all data has been synthesized from published literature.
